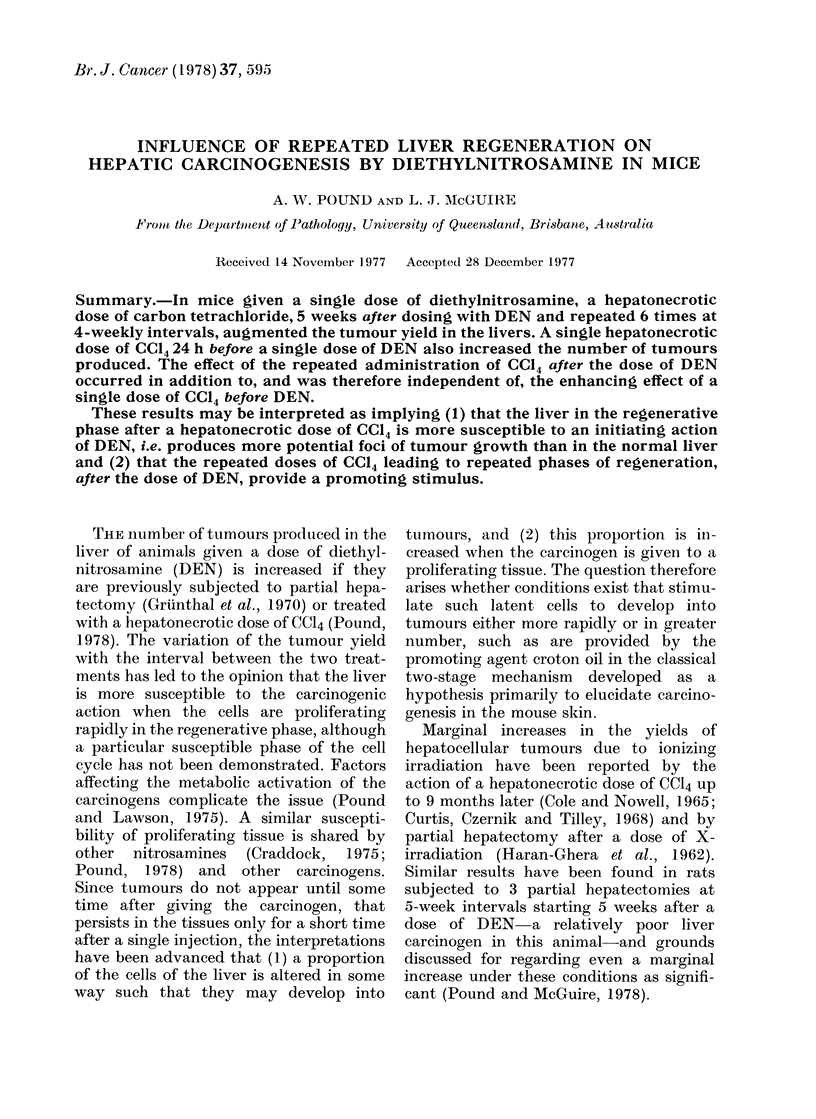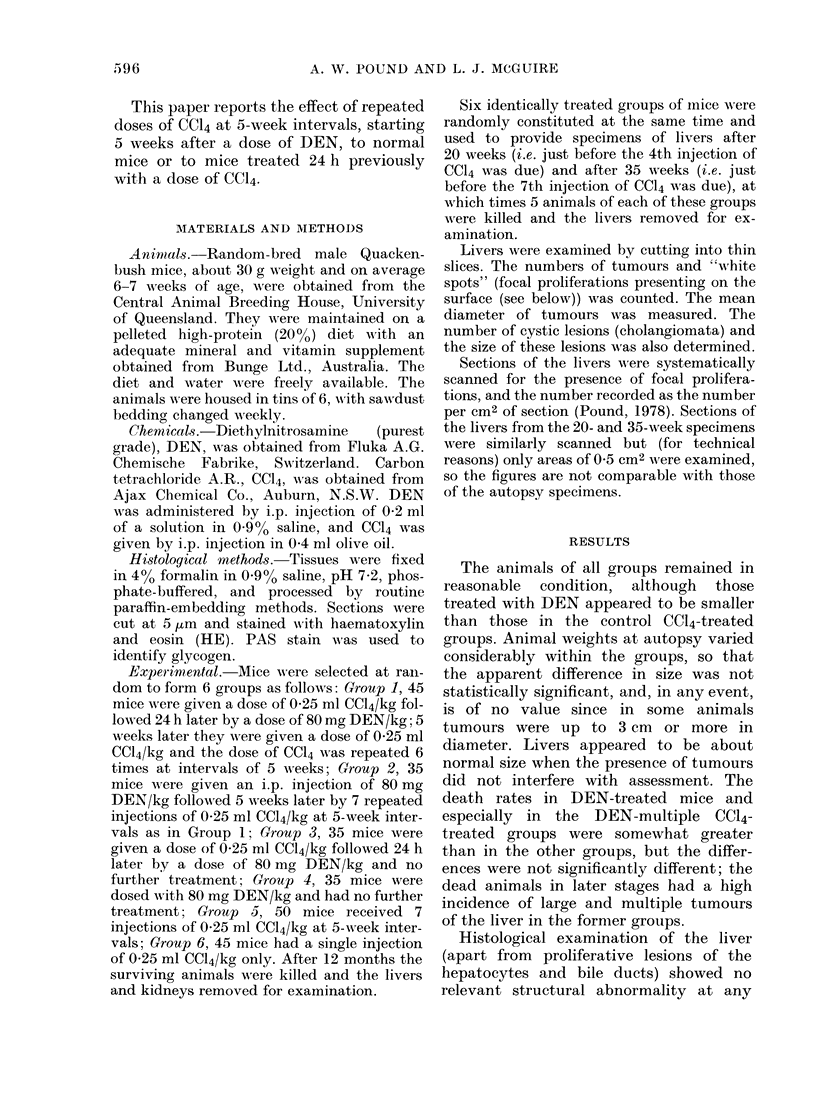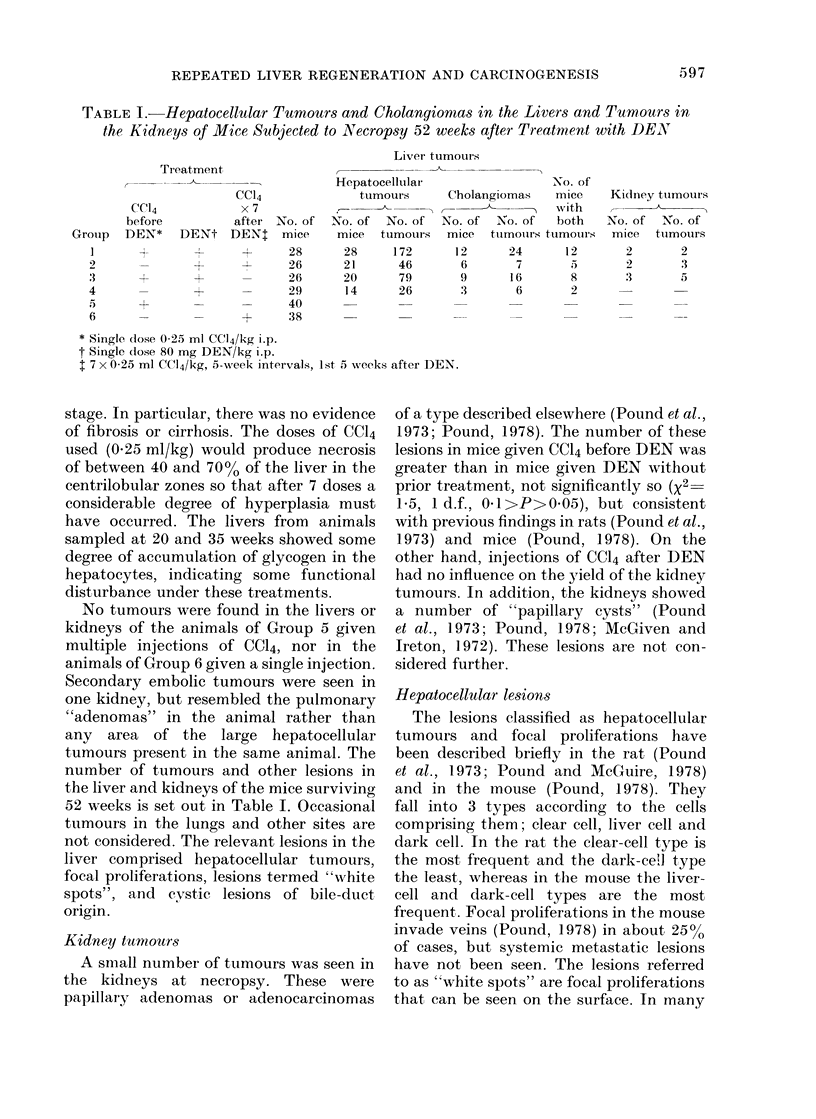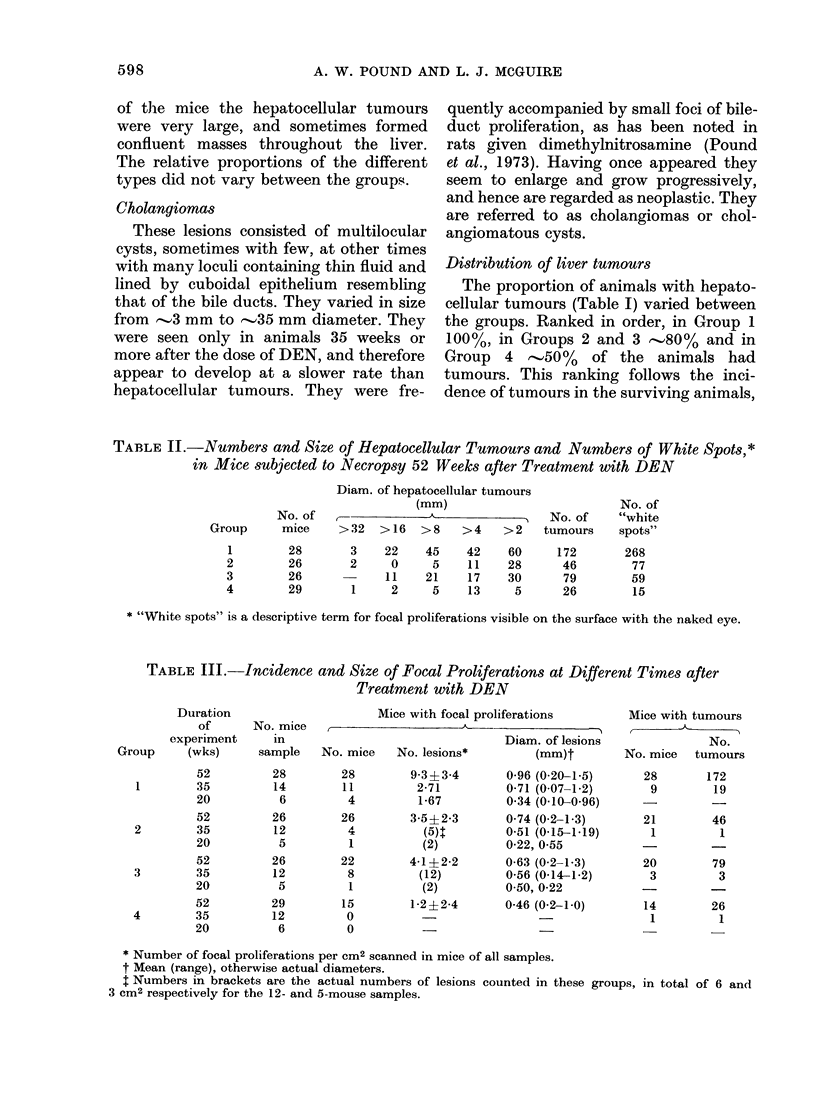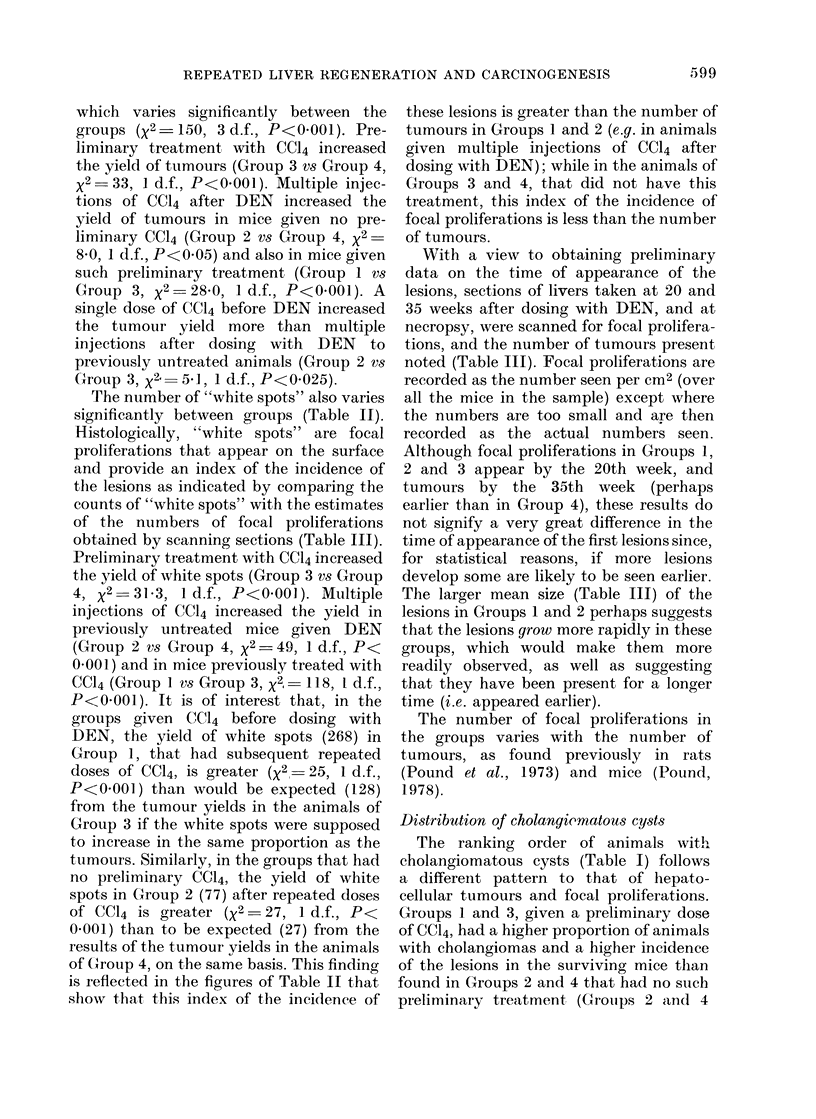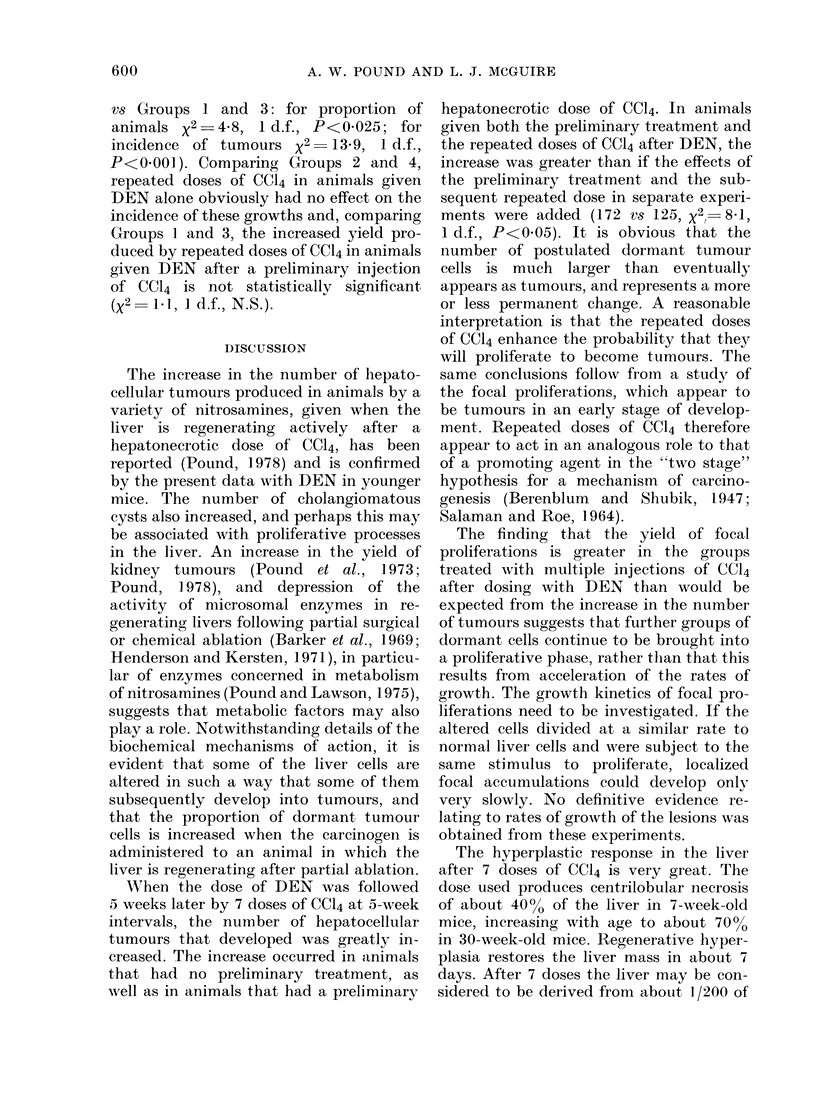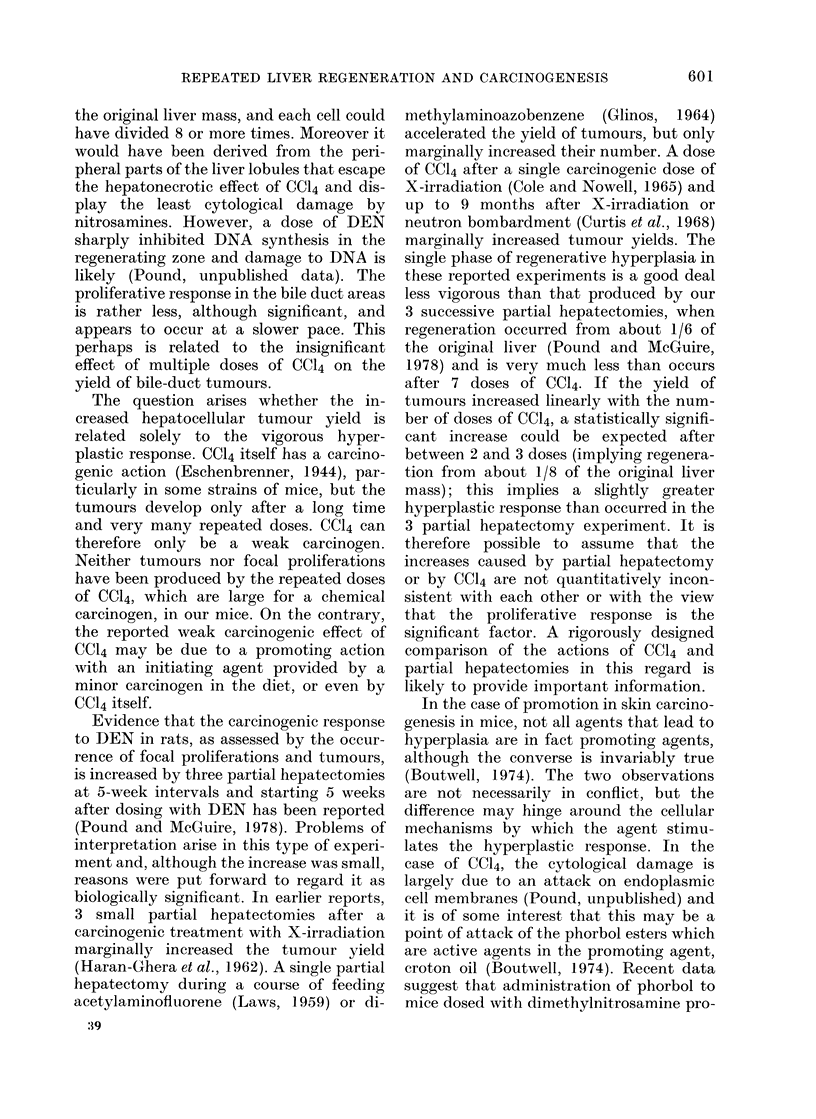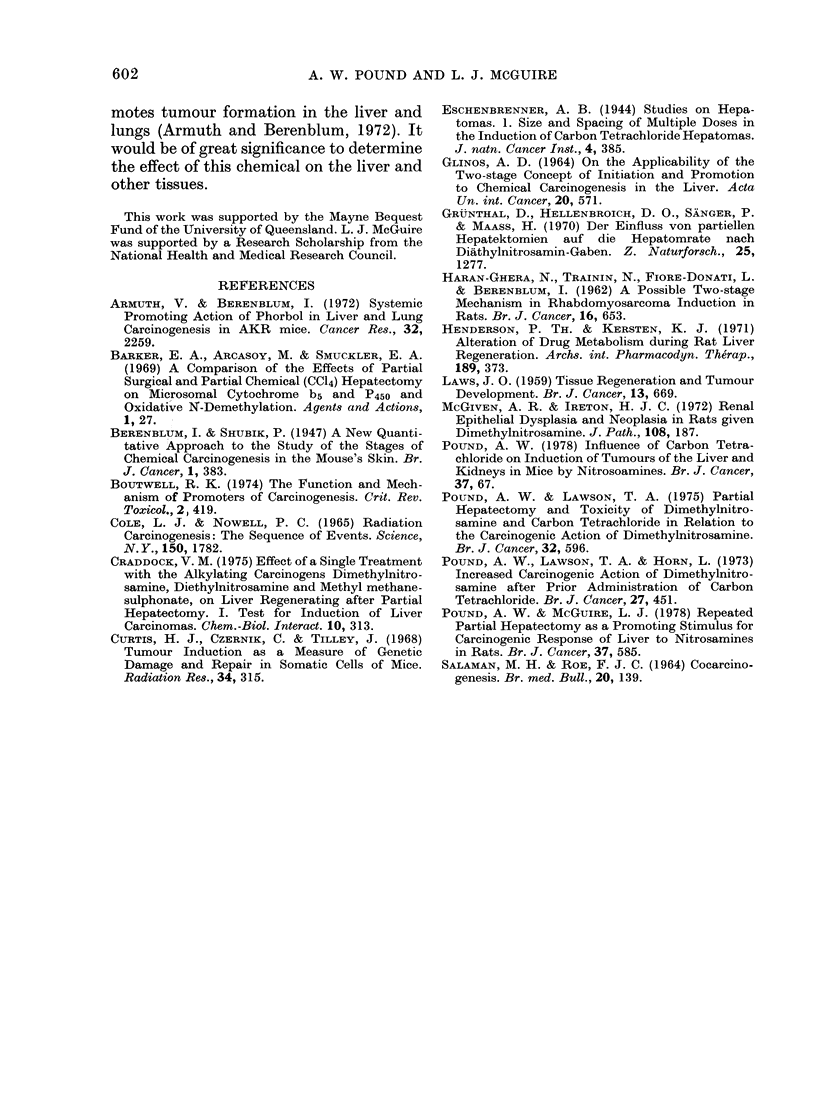# Influence of repeated liver regeneration on hepatic carcinogenesis by diethylnitrosamine in mice.

**DOI:** 10.1038/bjc.1978.89

**Published:** 1978-04

**Authors:** A. W. Poound, L. J. McGuire

## Abstract

In mice given a single dose of diethylnitrosamine, a hepatonecrotic dose of carbon tetrachloride, 5 weeks after dosing with DEN and repeated 6 times at 4-weekly intervals, augmented the tumour yield in the livers. A single hepatonecrotic dose of CCl4 24 h before a single dose of DEN also increased the number of tumours produced. The effect of the repeated administration of CCl4 after the dose of DEN occurred in addition to, and was therefore independent of, the enhancing effect of a single dose of CCl4 before DEN. These results may be interpreted as implying (1) that the liver in the regenerative phase after a hepatonecrotic dose of CCl4 is more susceptible to an initiating action of DEN, i.e. produces more potential foci of tumour growth than in the normal liver and (2) that the repeated doses of CCl4 leading to repeated phases of regeneration, after the dose of DEN, provide a promoting stimulus.


*Br. J. Cancer* (1978) **37**, 595

### INFLUENCE OF REPEATED LIVER REGENERATION ON HEPATIC CARCINOGENESIS BY DIETHYLNITROSAMINE IN MICE

A. W. POUND AND L. J. McGUIRE

*From the Department of Pathology, University of Queensland, Brisbane, Australia*

Received 14 November 1977 Accepted 28 December 1977

**Summary.—In mice given a single dose of diethylnitrosamine, a hepatonecrotic dose of carbon tetrachloride, 5 weeks *after* dosing with DEN and repeated 6 times at 4-weekly intervals, augmented the tumour yield in the livers. A single hepatonecrotic dose of $CCl_4$ 24 h *before* a single dose of DEN also increased the number of tumours produced. The effect of the repeated administration of $CCl_4$ *after* the dose of DEN occurred in addition to, and was therefore independent of, the enhancing effect of a single dose of $CCl_4$ *before* DEN.**

**These results may be interpreted as implying (1) that the liver in the regenerative phase after a hepatonecrotic dose of $CCl_4$ is more susceptible to an initiating action of DEN, *i.e.* produces more potential foci of tumour growth than in the normal liver and (2) that the repeated doses of $CCl_4$ leading to repeated phases of regeneration, *after* the dose of DEN, provide a promoting stimulus.**

THE number of tumours produced in the liver of animals given a dose of diethylnitrosamine (DEN) is increased if they are previously subjected to partial hepatectomy (Grünthal *et al.*, 1970) or treated with a hepatonecrotic dose of $CCl_4$ (Pound, 1978). The variation of the tumour yield with the interval between the two treatments has led to the opinion that the liver is more susceptible to the carcinogenic action when the cells are proliferating rapidly in the regenerative phase, although a particular susceptible phase of the cell cycle has not been demonstrated. Factors affecting the metabolic activation of the carcinogens complicate the issue (Pound and Lawson, 1975). A similar susceptibility of proliferating tissue is shared by other nitrosamines (Craddock, 1975; Pound, 1978) and other carcinogens. Since tumours do not appear until some time after giving the carcinogen, that persists in the tissues only for a short time after a single injection, the interpretations have been advanced that (1) a proportion of the cells of the liver is altered in some way such that they may develop into tumours, and (2) this proportion is increased when the carcinogen is given to a proliferating tissue. The question therefore arises whether conditions exist that stimulate such latent cells to develop into tumours either more rapidly or in greater number, such as are provided by the promoting agent croton oil in the classical two-stage mechanism developed as a hypothesis primarily to elucidate carcinogenesis in the mouse skin.

Marginal increases in the yields of hepatocellular tumours due to ionizing irradiation have been reported by the action of a hepatonecrotic dose of $CCl_4$ up to 9 months later (Cole and Nowell, 1965; Curtis, Czernik and Tilley, 1968) and by partial hepatectomy after a dose of X-irradiation (Haran-Ghera *et al.*, 1962). Similar results have been found in rats subjected to 3 partial hepatectomies at 5-week intervals starting 5 weeks after a dose of DEN—a relatively poor liver carcinogen in this animal—and grounds discussed for regarding even a marginal increase under these conditions as significant (Pound and McGuire, 1978).

This paper reports the effect of repeated doses of $CCl_4$ at 5-week intervals, starting 5 weeks after a dose of DEN, to normal mice or to mice treated 24 h previously with a dose of $CCl_4$.

### MATERIALS AND METHODS

*Animals.*—Random-bred male Quackenbush mice, about 30 g weight and on average 6–7 weeks of age, were obtained from the Central Animal Breeding House, University of Queensland. They were maintained on a pelleted high-protein (20%) diet with an adequate mineral and vitamin supplement obtained from Bunge Ltd., Australia. The diet and water were freely available. The animals were housed in tins of 6, with sawdust bedding changed weekly.

*Chemicals.*—Diethylnitrosamine (purest grade), DEN, was obtained from Fluka A.G. Chemische Fabrike, Switzerland. Carbon tetrachloride A.R., $CCl_4$, was obtained from Ajax Chemical Co., Auburn, N.S.W. DEN was administered by i.p. injection of 0·2 ml of a solution in 0·9% saline, and $CCl_4$ was given by i.p. injection in 0·4 ml olive oil.

*Histological methods.*—Tissues were fixed in 4% formalin in 0·9% saline, pH 7·2, phosphate-buffered, and processed by routine paraffin-embedding methods. Sections were cut at 5 $\mu$m and stained with haematoxylin and eosin (HE). PAS stain was used to identify glycogen.

*Experimental.*—Mice were selected at random to form 6 groups as follows: *Group 1*, 45 mice were given a dose of 0·25 ml $CCl_4$/kg followed 24 h later by a dose of 80 mg DEN/kg; 5 weeks later they were given a dose of 0·25 ml $CCl_4$/kg and the dose of $CCl_4$ was repeated 6 times at intervals of 5 weeks; *Group 2*, 35 mice were given an i.p. injection of 80 mg DEN/kg followed 5 weeks later by 7 repeated injections of 0·25 ml $CCl_4$/kg at 5-week intervals as in Group 1; *Group 3*, 35 mice were given a dose of 0·25 ml $CCl_4$/kg followed 24 h later by a dose of 80 mg DEN/kg and no further treatment; *Group 4*, 35 mice were dosed with 80 mg DEN/kg and had no further treatment; *Group 5*, 50 mice received 7 injections of 0·25 ml $CCl_4$/kg at 5-week intervals; *Group 6*, 45 mice had a single injection of 0·25 ml $CCl_4$/kg only. After 12 months the surviving animals were killed and the livers and kidneys removed for examination.

Six identically treated groups of mice were randomly constituted at the same time and used to provide specimens of livers after 20 weeks (*i.e.* just before the 4th injection of $CCl_4$ was due) and after 35 weeks (*i.e.* just before the 7th injection of $CCl_4$ was due), at which times 5 animals of each of these groups were killed and the livers removed for examination.

Livers were examined by cutting into thin slices. The numbers of tumours and "white spots" (focal proliferations presenting on the surface (see below)) was counted. The mean diameter of tumours was measured. The number of cystic lesions (cholangiomata) and the size of these lesions was also determined.

Sections of the livers were systematically scanned for the presence of focal proliferations, and the number recorded as the number per $cm^2$ of section (Pound, 1978). Sections of the livers from the 20- and 35-week specimens were similarly scanned but (for technical reasons) only areas of 0·5 $cm^2$ were examined, so the figures are not comparable with those of the autopsy specimens.

### RESULTS

The animals of all groups remained in reasonable condition, although those treated with DEN appeared to be smaller than those in the control $CCl_4$-treated groups. Animal weights at autopsy varied considerably within the groups, so that the apparent difference in size was not statistically significant, and, in any event, is of no value since in some animals tumours were up to 3 cm or more in diameter. Livers appeared to be about normal size when the presence of tumours did not interfere with assessment. The death rates in DEN-treated mice and especially in the DEN-multiple $CCl_4$-treated groups were somewhat greater than in the other groups, but the differences were not significantly different; the dead animals in later stages had a high incidence of large and multiple tumours of the liver in the former groups.

Histological examination of the liver (apart from proliferative lesions of the hepatocytes and bile ducts) showed no relevant structural abnormality at any

TABLE I.—*Hepatocellular Tumours and Cholangiomas in the Livers and Tumours in the Kidneys of Mice Subjected to Necropsy 52 weeks after Treatment with DEN*

| | Treatment | | | | Liver tumours | | | | | Kidney tumours | |
|---|---|---|---|---|---|---|---|---|---|---|---|
| | | | | | Hepatocellular tumours | | Cholangiomas | | | | |
| Group | $CCl_4$ before DEN* | DEN† | $CCl_4$ ×7 after DEN‡ | No. of mice | No. of mice | No. of tumours | No. of mice | No. of tumours | No. of mice with both tumours | No. of mice | No. of tumours |
| 1 | + | + | + | 28 | 28 | 172 | 12 | 24 | 12 | 2 | 2 |
| 2 | — | + | + | 26 | 21 | 46 | 6 | 7 | 5 | 2 | 3 |
| 3 | + | + | — | 26 | 20 | 79 | 9 | 16 | 8 | 3 | 5 |
| 4 | — | + | — | 29 | 14 | 26 | 3 | 6 | 2 | — | — |
| 5 | + | — | — | 40 | — | — | — | — | — | — | — |
| 6 | — | — | + | 38 | — | — | — | — | — | — | — |

* Single dose 0·25 ml $CCl_4$/kg i.p.

† Single dose 80 mg DEN/kg i.p.

‡ 7 × 0·25 ml $CCl_4$/kg, 5-week intervals, 1st 5 weeks after DEN.

stage. In particular, there was no evidence of fibrosis or cirrhosis. The doses of $CCl_4$ used (0·25 ml/kg) would produce necrosis of between 40 and 70% of the liver in the centrilobular zones so that after 7 doses a considerable degree of hyperplasia must have occurred. The livers from animals sampled at 20 and 35 weeks showed some degree of accumulation of glycogen in the hepatocytes, indicating some functional disturbance under these treatments.

No tumours were found in the livers or kidneys of the animals of Group 5 given multiple injections of $CCl_4$, nor in the animals of Group 6 given a single injection. Secondary embolic tumours were seen in one kidney, but resembled the pulmonary "adenomas" in the animal rather than any area of the large hepatocellular tumours present in the same animal. The number of tumours and other lesions in the liver and kidneys of the mice surviving 52 weeks is set out in Table I. Occasional tumours in the lungs and other sites are not considered. The relevant lesions in the liver comprised hepatocellular tumours, focal proliferations, lesions termed "white spots", and cystic lesions of bile-duct origin.

*Kidney tumours*

A small number of tumours was seen in the kidneys at necropsy. These were papillary adenomas or adenocarcinomas of a type described elsewhere (Pound *et al.*, 1973; Pound, 1978). The number of these lesions in mice given $CCl_4$ before DEN was greater than in mice given DEN without prior treatment, not significantly so ($\chi^2 =$ 1·5, 1 d.f., $0{\cdot}1 > P > 0{\cdot}05$), but consistent with previous findings in rats (Pound *et al.*, 1973) and mice (Pound, 1978). On the other hand, injections of $CCl_4$ after DEN had no influence on the yield of the kidney tumours. In addition, the kidneys showed a number of "papillary cysts" (Pound *et al.*, 1973; Pound, 1978; McGiven and Ireton, 1972). These lesions are not considered further.

*Hepatocellular lesions*

The lesions classified as hepatocellular tumours and focal proliferations have been described briefly in the rat (Pound *et al.*, 1973; Pound and McGuire, 1978) and in the mouse (Pound, 1978). They fall into 3 types according to the cells comprising them; clear cell, liver cell and dark cell. In the rat the clear-cell type is the most frequent and the dark-cell type the least, whereas in the mouse the liver-cell and dark-cell types are the most frequent. Focal proliferations in the mouse invade veins (Pound, 1978) in about 25% of cases, but systemic metastatic lesions have not been seen. The lesions referred to as "white spots" are focal proliferations that can be seen on the surface. In many of the mice the hepatocellular tumours were very large, and sometimes formed confluent masses throughout the liver. The relative proportions of the different types did not vary between the groups.

*Cholangiomas*

These lesions consisted of multilocular cysts, sometimes with few, at other times with many loculi containing thin fluid and lined by cuboidal epithelium resembling that of the bile ducts. They varied in size from ~3 mm to ~35 mm diameter. They were seen only in animals 35 weeks or more after the dose of DEN, and therefore appear to develop at a slower rate than hepatocellular tumours. They were frequently accompanied by small foci of bile-duct proliferation, as has been noted in rats given dimethylnitrosamine (Pound *et al.*, 1973). Having once appeared they seem to enlarge and grow progressively, and hence are regarded as neoplastic. They are referred to as cholangiomas or cholangiomatous cysts.

*Distribution of liver tumours*

The proportion of animals with hepatocellular tumours (Table I) varied between the groups. Ranked in order, in Group 1 100%, in Groups 2 and 3 ~80% and in Group 4 ~50% of the animals had tumours. This ranking follows the incidence of tumours in the surviving animals,

TABLE II.—*Numbers and Size of Hepatocellular Tumours and Numbers of White Spots,* in Mice subjected to Necropsy 52 Weeks after Treatment with DEN*

| Group | No. of mice | Diam. of hepatocellular tumours (mm) >32 | >16 | >8 | >4 | >2 | No. of tumours | No. of "white spots" |
|---|---|---|---|---|---|---|---|---|
| 1 | 28 | 3 | 22 | 45 | 42 | 60 | 172 | 268 |
| 2 | 26 | 2 | 0 | 5 | 11 | 28 | 46 | 77 |
| 3 | 26 | — | 11 | 21 | 17 | 30 | 79 | 59 |
| 4 | 29 | 1 | 2 | 5 | 13 | 5 | 26 | 15 |

* "White spots" is a descriptive term for focal proliferations visible on the surface with the naked eye.

TABLE III.—*Incidence and Size of Focal Proliferations at Different Times after Treatment with DEN*

| Group | Duration of experiment (wks) | No. mice in sample | Mice with focal proliferations: No. mice | No. lesions* | Diam. of lesions (mm)† | Mice with tumours: No. mice | No. tumours |
|---|---|---|---|---|---|---|---|
| | 52 | 28 | 28 | 9·3±3·4 | 0·96 (0·20–1·5) | 28 | 172 |
| 1 | 35 | 14 | 11 | 2·71 | 0·71 (0·07–1·2) | 9 | 19 |
| | 20 | 6 | 4 | 1·67 | 0·34 (0·10–0·96) | — | — |
| | 52 | 26 | 26 | 3·5±2·3 | 0·74 (0·2–1·3) | 21 | 46 |
| 2 | 35 | 12 | 4 | (5)‡ | 0·51 (0·15–1·19) | 1 | 1 |
| | 20 | 5 | 1 | (2) | 0·22, 0·55 | — | — |
| | 52 | 26 | 22 | 4·1±2·2 | 0·63 (0·2–1·3) | 20 | 79 |
| 3 | 35 | 12 | 8 | (12) | 0·56 (0·14–1·2) | 3 | 3 |
| | 20 | 5 | 1 | (2) | 0·50, 0·22 | — | — |
| | 52 | 29 | 15 | 1·2±2·4 | 0·46 (0·2–1·0) | 14 | 26 |
| 4 | 35 | 12 | 0 | — | — | 1 | 1 |
| | 20 | 6 | 0 | — | — | — | — |

* Number of focal proliferations per cm² scanned in mice of all samples.
† Mean (range), otherwise actual diameters.
‡ Numbers in brackets are the actual numbers of lesions counted in these groups, in total of 6 and 3 cm² respectively for the 12- and 5-mouse samples.

which varies significantly between the groups ($\chi^2 = 150$, 3 d.f., $P < 0{\cdot}001$). Preliminary treatment with $CCl_4$ increased the yield of tumours (Group 3 *vs* Group 4, $\chi^2 = 33$, 1 d.f., $P < 0{\cdot}001$). Multiple injections of $CCl_4$ after DEN increased the yield of tumours in mice given no preliminary $CCl_4$ (Group 2 *vs* Group 4, $\chi^2 = 8{\cdot}0$, 1 d.f., $P < 0{\cdot}05$) and also in mice given such preliminary treatment (Group 1 *vs* Group 3, $\chi^2 = 28{\cdot}0$, 1 d.f., $P < 0{\cdot}001$). A single dose of $CCl_4$ before DEN increased the tumour yield more than multiple injections after dosing with DEN to previously untreated animals (Group 2 *vs* Group 3, $\chi^2 = 5{\cdot}1$, 1 d.f., $P < 0{\cdot}025$).

The number of "white spots" also varies significantly between groups (Table II). Histologically, "white spots" are focal proliferations that appear on the surface and provide an index of the incidence of the lesions as indicated by comparing the counts of "white spots" with the estimates of the numbers of focal proliferations obtained by scanning sections (Table III). Preliminary treatment with $CCl_4$ increased the yield of white spots (Group 3 *vs* Group 4, $\chi^2 = 31{\cdot}3$, 1 d.f., $P < 0{\cdot}001$). Multiple injections of $CCl_4$ increased the yield in previously untreated mice given DEN (Group 2 *vs* Group 4, $\chi^2 = 49$, 1 d.f., $P < 0{\cdot}001$) and in mice previously treated with $CCl_4$ (Group 1 *vs* Group 3, $\chi^2 = 118$, 1 d.f., $P < 0{\cdot}001$). It is of interest that, in the groups given $CCl_4$ before dosing with DEN, the yield of white spots (268) in Group 1, that had subsequent repeated doses of $CCl_4$, is greater ($\chi^2 = 25$, 1 d.f., $P < 0{\cdot}001$) than would be expected (128) from the tumour yields in the animals of Group 3 if the white spots were supposed to increase in the same proportion as the tumours. Similarly, in the groups that had no preliminary $CCl_4$, the yield of white spots in Group 2 (77) after repeated doses of $CCl_4$ is greater ($\chi^2 = 27$, 1 d.f., $P < 0{\cdot}001$) than to be expected (27) from the results of the tumour yields in the animals of Group 4, on the same basis. This finding is reflected in the figures of Table II that show that this index of the incidence of these lesions is greater than the number of tumours in Groups 1 and 2 (*e.g.* in animals given multiple injections of $CCl_4$ after dosing with DEN); while in the animals of Groups 3 and 4, that did not have this treatment, this index of the incidence of focal proliferations is less than the number of tumours.

With a view to obtaining preliminary data on the time of appearance of the lesions, sections of livers taken at 20 and 35 weeks after dosing with DEN, and at necropsy, were scanned for focal proliferations, and the number of tumours present noted (Table III). Focal proliferations are recorded as the number seen per $cm^2$ (over all the mice in the sample) except where the numbers are too small and are then recorded as the actual numbers seen. Although focal proliferations in Groups 1, 2 and 3 appear by the 20th week, and tumours by the 35th week (perhaps earlier than in Group 4), these results do not signify a very great difference in the time of appearance of the first lesions since, for statistical reasons, if more lesions develop some are likely to be seen earlier. The larger mean size (Table III) of the lesions in Groups 1 and 2 perhaps suggests that the lesions *grow* more rapidly in these groups, which would make them more readily observed, as well as suggesting that they have been present for a longer time (*i.e.* appeared earlier).

The number of focal proliferations in the groups varies with the number of tumours, as found previously in rats (Pound *et al.*, 1973) and mice (Pound, 1978).

*Distribution of cholangiomatous cysts*

The ranking order of animals with cholangiomatous cysts (Table I) follows a different pattern to that of hepatocellular tumours and focal proliferations. Groups 1 and 3, given a preliminary dose of $CCl_4$, had a higher proportion of animals with cholangiomas and a higher incidence of the lesions in the surviving mice than found in Groups 2 and 4 that had no such preliminary treatment (Groups 2 and 4

*vs* Groups 1 and 3: for proportion of animals $\chi^2 = 4{\cdot}8$, 1 d.f., $P < 0{\cdot}025$; for incidence of tumours $\chi^2 = 13{\cdot}9$, 1 d.f., $P < 0{\cdot}001$). Comparing Groups 2 and 4, repeated doses of $CCl_4$ in animals given DEN alone obviously had no effect on the incidence of these growths and, comparing Groups 1 and 3, the increased yield produced by repeated doses of $CCl_4$ in animals given DEN after a preliminary injection of $CCl_4$ is not statistically significant ($\chi^2 = 1{\cdot}1$, 1 d.f., N.S.).

### DISCUSSION

The increase in the number of hepatocellular tumours produced in animals by a variety of nitrosamines, given when the liver is regenerating actively after a hepatonecrotic dose of $CCl_4$, has been reported (Pound, 1978) and is confirmed by the present data with DEN in younger mice. The number of cholangiomatous cysts also increased, and perhaps this may be associated with proliferative processes in the liver. An increase in the yield of kidney tumours (Pound *et al.*, 1973; Pound, 1978), and depression of the activity of microsomal enzymes in regenerating livers following partial surgical or chemical ablation (Barker *et al.*, 1969; Henderson and Kersten, 1971), in particular of enzymes concerned in metabolism of nitrosamines (Pound and Lawson, 1975), suggests that metabolic factors may also play a role. Notwithstanding details of the biochemical mechanisms of action, it is evident that some of the liver cells are altered in such a way that some of them subsequently develop into tumours, and that the proportion of dormant tumour cells is increased when the carcinogen is administered to an animal in which the liver is regenerating after partial ablation.

When the dose of DEN was followed 5 weeks later by 7 doses of $CCl_4$ at 5-week intervals, the number of hepatocellular tumours that developed was greatly increased. The increase occurred in animals that had no preliminary treatment, as well as in animals that had a preliminary hepatonecrotic dose of $CCl_4$. In animals given both the preliminary treatment and the repeated doses of $CCl_4$ after DEN, the increase was greater than if the effects of the preliminary treatment and the subsequent repeated dose in separate experiments were added (172 *vs* 125, $\chi^2 = 8{\cdot}1$, 1 d.f., $P < 0{\cdot}05$). It is obvious that the number of postulated dormant tumour cells is much larger than eventually appears as tumours, and represents a more or less permanent change. A reasonable interpretation is that the repeated doses of $CCl_4$ enhance the probability that they will proliferate to become tumours. The same conclusions follow from a study of the focal proliferations, which appear to be tumours in an early stage of development. Repeated doses of $CCl_4$ therefore appear to act in an analogous role to that of a promoting agent in the "two stage" hypothesis for a mechanism of carcinogenesis (Berenblum and Shubik, 1947; Salaman and Roe, 1964).

The finding that the yield of focal proliferations is greater in the groups treated with multiple injections of $CCl_4$ after dosing with DEN than would be expected from the increase in the number of tumours suggests that further groups of dormant cells continue to be brought into a proliferative phase, rather than that this results from acceleration of the rates of growth. The growth kinetics of focal proliferations need to be investigated. If the altered cells divided at a similar rate to normal liver cells and were subject to the same stimulus to proliferate, localized focal accumulations could develop only very slowly. No definitive evidence relating to rates of growth of the lesions was obtained from these experiments.

The hyperplastic response in the liver after 7 doses of $CCl_4$ is very great. The dose used produces centrilobular necrosis of about 40% of the liver in 7-week-old mice, increasing with age to about 70% in 30-week-old mice. Regenerative hyperplasia restores the liver mass in about 7 days. After 7 doses the liver may be considered to be derived from about 1/200 of the original liver mass, and each cell could have divided 8 or more times. Moreover it would have been derived from the peripheral parts of the liver lobules that escape the hepatonecrotic effect of $CCl_4$ and display the least cytological damage by nitrosamines. However, a dose of DEN sharply inhibited DNA synthesis in the regenerating zone and damage to DNA is likely (Pound, unpublished data). The proliferative response in the bile duct areas is rather less, although significant, and appears to occur at a slower pace. This perhaps is related to the insignificant effect of multiple doses of $CCl_4$ on the yield of bile-duct tumours.

The question arises whether the increased hepatocellular tumour yield is related solely to the vigorous hyperplastic response. $CCl_4$ itself has a carcinogenic action (Eschenbrenner, 1944), particularly in some strains of mice, but the tumours develop only after a long time and very many repeated doses. $CCl_4$ can therefore only be a weak carcinogen. Neither tumours nor focal proliferations have been produced by the repeated doses of $CCl_4$, which are large for a chemical carcinogen, in our mice. On the contrary, the reported weak carcinogenic effect of $CCl_4$ may be due to a promoting action with an initiating agent provided by a minor carcinogen in the diet, or even by $CCl_4$ itself.

Evidence that the carcinogenic response to DEN in rats, as assessed by the occurrence of focal proliferations and tumours, is increased by three partial hepatectomies at 5-week intervals and starting 5 weeks after dosing with DEN has been reported (Pound and McGuire, 1978). Problems of interpretation arise in this type of experiment and, although the increase was small, reasons were put forward to regard it as biologically significant. In earlier reports, 3 small partial hepatectomies after a carcinogenic treatment with X-irradiation marginally increased the tumour yield (Haran-Ghera *et al.*, 1962). A single partial hepatectomy during a course of feeding acetylaminofluorene (Laws, 1959) or dimethylaminoazobenzene (Glinos, 1964) accelerated the yield of tumours, but only marginally increased their number. A dose of $CCl_4$ after a single carcinogenic dose of X-irradiation (Cole and Nowell, 1965) and up to 9 months after X-irradiation or neutron bombardment (Curtis *et al.*, 1968) marginally increased tumour yields. The single phase of regenerative hyperplasia in these reported experiments is a good deal less vigorous than that produced by our 3 successive partial hepatectomies, when regeneration occurred from about 1/6 of the original liver (Pound and McGuire, 1978) and is very much less than occurs after 7 doses of $CCl_4$. If the yield of tumours increased linearly with the number of doses of $CCl_4$, a statistically significant increase could be expected after between 2 and 3 doses (implying regeneration from about 1/8 of the original liver mass); this implies a slightly greater hyperplastic response than occurred in the 3 partial hepatectomy experiment. It is therefore possible to assume that the increases caused by partial hepatectomy or by $CCl_4$ are not quantitatively inconsistent with each other or with the view that the proliferative response is the significant factor. A rigorously designed comparison of the actions of $CCl_4$ and partial hepatectomies in this regard is likely to provide important information.

In the case of promotion in skin carcinogenesis in mice, not all agents that lead to hyperplasia are in fact promoting agents, although the converse is invariably true (Boutwell, 1974). The two observations are not necessarily in conflict, but the difference may hinge around the cellular mechanisms by which the agent stimulates the hyperplastic response. In the case of $CCl_4$, the cytological damage is largely due to an attack on endoplasmic cell membranes (Pound, unpublished) and it is of some interest that this may be a point of attack of the phorbol esters which are active agents in the promoting agent, croton oil (Boutwell, 1974). Recent data suggest that administration of phorbol to mice dosed with dimethylnitrosamine promotes tumour formation in the liver and lungs (Armuth and Berenblum, 1972). It would be of great significance to determine the effect of this chemical on the liver and other tissues.

This work was supported by the Mayne Bequest Fund of the University of Queensland. L. J. McGuire was supported by a Research Scholarship from the National Health and Medical Research Council.

### REFERENCES

ARMUTH, V. & BERENBLUM, I. (1972) Systemic Promoting Action of Phorbol in Liver and Lung Carcinogenesis in AKR mice. *Cancer Res.*, **32**, 2259.

BARKER, E. A., ARCASOY, M. & SMUCKLER, E. A. (1969) A Comparison of the Effects of Partial Surgical and Partial Chemical ($CCl_4$) Hepatectomy on Microsomal Cytochrome $b_5$ and $P_{450}$ and Oxidative N-Demethylation. *Agents and Actions*, **1**, 27.

BERENBLUM, I. & SHUBIK, P. (1947) A New Quantitative Approach to the Study of the Stages of Chemical Carcinogenesis in the Mouse's Skin. *Br. J. Cancer*, **1**, 383.

BOUTWELL, R. K. (1974) The Function and Mechanism of Promoters of Carcinogenesis. *Crit. Rev. Toxicol.*, **2**, 419.

COLE, L. J. & NOWELL, P. C. (1965) Radiation Carcinogenesis: The Sequence of Events. *Science, N.Y.*, **150**, 1782.

CRADDOCK, V. M. (1975) Effect of a Single Treatment with the Alkylating Carcinogens Dimethylnitrosamine, Diethylnitrosamine and Methyl methanesulphonate, on Liver Regenerating after Partial Hepatectomy. I. Test for Induction of Liver Carcinomas. *Chem.-Biol. Interact.* **10**, 313.

CURTIS, H. J., CZERNIK, C. & TILLEY, J. (1968) Tumour Induction as a Measure of Genetic Damage and Repair in Somatic Cells of Mice. *Radiation Res.*, **34**, 315.

ESCHENBRENNER, A. B. (1944) Studies on Hepatomas. 1. Size and Spacing of Multiple Doses in the Induction of Carbon Tetrachloride Hepatomas. *J. natn. Cancer Inst.*, **4**, 385.

GLINOS, A. D. (1964) On the Applicability of the Two-stage Concept of Initiation and Promotion to Chemical Carcinogenesis in the Liver. *Acta Un. int. Cancer*, **20**, 571.

GRÜNTHAL, D., HELLENBROICH, D. O., SÄNGER, P. & MAASS, H. (1970) Der Einfluss von partiellen Hepatektomien auf die Hepatomrate nach Diäthylnitrosamin-Gaben. *Z. Naturforsch.*, **25**, 1277.

HARAN-GHERA, N., TRAININ, N., FIORE-DONATI, L. & BERENBLUM, I. (1962) A Possible Two-stage Mechanism in Rhabdomyosarcoma Induction in Rats. *Br. J. Cancer*, **16**, 653.

HENDERSON, P. TH. & KERSTEN, K. J. (1971) Alteration of Drug Metabolism during Rat Liver Regeneration. *Archs. int. Pharmacodyn. Thérap.*, **189**, 373.

LAWS, J. O. (1959) Tissue Regeneration and Tumour Development. *Br. J. Cancer*, **13**, 669.

MCGIVEN, A. R. & IRETON, H. J. C. (1972) Renal Epithelial Dysplasia and Neoplasia in Rats given Dimethylnitrosamine. *J. Path.*, **108**, 187.

POUND, A. W. (1978) Influence of Carbon Tetrachloride on Induction of Tumours of the Liver and Kidneys in Mice by Nitrosoamines. *Br. J. Cancer*, **37**, 67.

POUND, A. W. & LAWSON, T. A. (1975) Partial Hepatectomy and Toxicity of Dimethylnitrosamine and Carbon Tetrachloride in Relation to the Carcinogenic Action of Dimethylnitrosamine. *Br. J. Cancer*, **32**, 596.

POUND, A. W., LAWSON, T. A. & HORN, L. (1973) Increased Carcinogenic Action of Dimethylnitrosamine after Prior Administration of Carbon Tetrachloride. *Br. J. Cancer*, **27**, 451.

POUND, A. W. & MCGUIRE, L. J. (1978) Repeated Partial Hepatectomy as a Promoting Stimulus for Carcinogenic Response of Liver to Nitrosamines in Rats. *Br. J. Cancer*, **37**, 585.

SALAMAN, M. H. & ROE, F. J. C. (1964) Cocarcinogenesis. *Br. med. Bull.*, **20**, 139.